# Effects of Adding Legume Flours on the Rheological and Breadmaking Properties of Dough

**DOI:** 10.3390/foods10051087

**Published:** 2021-05-14

**Authors:** Tatiana Bojňanská, Janette Musilová, Alena Vollmannová

**Affiliations:** 1 Department of Technology and Quality of Plant Products, Faculty of Biotechnology and Food Sciences, Slovak University of Agriculture in Nitra, Trieda A. Hlinku 2, 949 76 Nitra, Slovakia; 2 Department of Chemistry, Faculty of Biotechnology and Food Sciences, Slovak University of Agriculture in Nitra, Trieda A. Hlinku 2, 949 76 Nitra, Slovakia; janette.musilova@uniag.sk (J.M.); alena.vollmannova@uniag.sk (A.V.)

**Keywords:** wheat-rye flour, chickpea flour, common bean flour, red lentil flour, broad bean flour, Mixolab, dough rheology, Rheofermentometer, gas retention, baking test, loaf volume, sensory evaluation

## Abstract

The influence of the addition of four legume flours, chickpea, broad bean, common bean and red lentil (in amounts of 5%, 10% and 15% to a wheat-rye composite flour (50:50:0-control flour), in ratios of 50:45:5; 50:40:10; 50:35:15) was studied by analyzing the rheological properties of dough in order to further exploit the functionality of legume flours in bakery products. The rheological properties of dough were monitored using a Mixolab 2. A Rheofermentometer F4 was used to check the dough fermentation, and a Volscan was used for evaluating the baking trials. The addition of different legume flours in the mixtures resulted in different viscoelastic properties of the dough. The results showed a weakening of the protein network depending on the amount of legume flour added and on the specific legume flour. On the contrary, all samples with a higher proportion of legume flour showed an increased resistance to starch retrogradation. All flours had the ability to produce a sufficient volume of fermenting gases, with the exception of flours with a higher addition of broad bean flour, and the baking test confirmed a lower bread volume for bread with this addition. The results of the sensory evaluation indicated that legume flour additions resulted in breads with an acceptable sensory quality, in the case of additions of 5% at the same level as the bread controls, or even better. The aromas and flavors of the added non-cereal ingredients improved the sensory profile of wheat-rye bread. Breads with additions of chickpea, common bean and broad bean had a considerable proportion of darker colors in comparison to the control bread and bread with red lentil.

## 1. Introduction

Bread has been part of the human diet for thousands of years and it remains the most regularly consumed food in the world, due to its convenience, portability, nutrition, and taste [1,2]. Bread is an important staple food with a traditional composition, and thus a given nutritional quality, typical of specific areas. However, some bread can be less nutritionally attractive [3,4]. The main raw material used worldwide for bread production is wheat flour, which is characterized mainly by a high proportion of starch and a number of proteins poor in essential amino acids. However, wheat flour is the only cereal flour that can form a three-dimensional viscoelastic dough when mixed with water. This unique ability of wheat to suit the production of leavened products is thanks to gluten, a cohesive, viscoelastic proteinaceous material, which retains fermenting gases. In Slovakia, the second traditional bakery raw material is rye flour, which has different technological properties, namely, it does not form gluten structures, and thus has a limited ability to create products with a large volume and good porosity [5,6,7].

Efforts have been made to improve the nutritional quality of bread. One of the possibilities is partial replacement of wheat with non-bakery raw materials, which are valued thanks to the content of ingredients that do not appear in traditional breads. This creates diverse products with beneficial health effects [8,9,10]. An important agricultural group of such raw materials are legumes [11], which are a valuable source of protein with a balanced amino acid profile, carbohydrates, fiber, vitamins, minerals and phytochemicals with a positive effect on health [12,13,14,15,16,17,18,19].

Regardless of the type of legumes, the substitution of wheat flour with legume flour considerably improves the nutritional value by increasing the protein, mineral and fiber content of bread, and decreasing the glycemic index [13,14,20,21,22,23]. However, in this type of flour the absence of gluten causes problems related to the technological properties of flour, dough and the final product due to contrasting differences between proteins, such as water solubility, differences in primary structure and size distribution, properties which are characteristic of the viscoelastic properties of wheat gluten. The addition of non-gluten-forming proteins causes dilution and subsequent weakening of the wheat dough. Legume proteins are unable to form gluten networks. The weak interactions between legume and wheat proteins reduce the dough viscoelasticity and affect the air incorporation and gas retention during fermentation, resulting in bread with a poor crumb structure and texture [9,10,14,20]. Nevertheless, fortifying wheat flour with legumes in order to develop new healthy food products might be the right trend to follow. In connection with the environmental sustainability of agriculture, legumes are also a source of low-carbon and low-water proteins. Bread made from mixtures containing legumes has an improved nutritional composition and satisfactory technological and sensory properties [4,19,24].

The suitability of alternative crops for the production of quality bread is examined in particular by measuring the properties of their mixtures with wheat or some other flour. The dough components (starch, proteins, and water) and their interactions play an important role in the conformational structure as well as in the rheological properties [9,20,25]. To characterize the dough rheology, a mixograph is used as an empirical tool to record mechanical changes due to mixing and heating, simulating the mechanical work as well as the thermal conditions that can be expected in the bread making and baking process. The advantage of using a Mixolab is that the properties of proteins and starch (and related enzymes) can be measured in one test [26,27,28]. The functionality of the gluten network developed through mixing is crucial for gas retention and the final structure of bread. During proofing, the rate of gas production depends on the activity of the baker’s yeast (*Saccharomyces cerevisiae)* and the gluten network. The rheofermentometer analysis is useful in predicting the real proofing performance of bread dough. This is used to estimate the dough properties during fermentation by measuring the released CO_2_ or produced pressure, considering that the produced CO_2_ serves to expand the dough and achieve the final bread loaf volume [29,30]. The baking test is the direct method of determining the quality of the applied raw materials and composite flours. During the baking process, the flour compounds are subjected to mechanical work and heat treatment that promote changes in their physicochemical properties [31]. The final product has physical and sensory properties that make it a well-digested and popular staple food.

The main aim of this research was to model some experimental doughs containing different levels of wheat flour, rye flour and legume flour (to enhance the nutritional benefits), in order to study the influence of the different ingredients on the rheological properties of the dough, its ability to retain fermentation gases, and the objective properties of the final product.

## 2. Materials and Methods

### 2.1. Material Used

In this study, commercial flours (Miroslav Grznár MLYN ZRNO, Veľké Hoste, Slovak Republic), were used namely WF (wheat flour), RF (rye flour), ChF (chickpea flour), BBF (broad bean flour), CBF (common bean flour), and RLF (red lentil flour). These commercially available flours have defined quality parameters (Table 1). According to the manufacturer, WF and RF had an ash content of 0.65% and 0.96%, respectively.

The selected tested legumes represent the widely used varieties found to be nutritionally enriching according to recent publications.

All other ingredients such as salt (K.S. Czech Republic, a. s.), sucrose (Slovenské cukrovary s.r.o.), and dry yeast of the species *Saccharomyces cerevisiae* (Ruf, sušené droždie) were purchased from the local market.

### 2.2. Preparation of Composite Flours

Three types of mixture of wheat flour and rye flour and different proportions of four legume flours, as well as a control flour without legume flour, were prepared in the following ratios: 50:45:5, 50:40:10, 50:35:15, and 50:50:0, respectively. The proportion of wheat flour in composite flours did not change (50% in each sample), but the mutual ratios of rye flour and legume flours varied as indicated in Table 2.

### 2.3. Mixolab Measurements

The mixing and pasting behavior of the composite flours were determined in a Mixolab 2 (Chopin Technologies, France) according to the ICC Standard Method 173 [32] using the “Chopin S” and “Chopin+” protocol (Table 3). The evaluated parameters from the mixolab curves were: water absorption (WA); dough development time (DDT); stability (mixing resistance of dough); C1 (maximum torque during mixing); C1.2 (torque at the end of the holding time at 30 °C); C2 (weakening of the protein, based on mechanical stress at increasing temperature); C3 (rate of starch gelatinization); C4 (minimum torque during the heating period); C5 (torque after cooling at 50 °C); alpha slope (protein weakening speed under heating effect); beta slope (speed of starch gelatinization); gamma slope (speed of enzyme degradation); C4/C3 (cooking stability), C5-C4 (starch retrogradation at cooling stage, representing the shelf-life of the end products). Mixolab software also offers a profiler function that simplifies data and outputs by processing large data. Although it is not considered primarily as a research tool, it is in a way a converter that converts complex information into simple quality indices. 

### 2.4. Rheofermentometer Analysis

A Rheofermentometer F4 (Chopin Technologies, France) was applied to detect the gas release kinetics depending on the varied quantities of legume flours. Immediately after kneading, 315 g of a biologically leavened dough sample was placed into the fermentation basket and was covered with the optical sensor. The proofing chamber was closed hermetically to begin the measuring series at 28.5 °C for 180 min. The following dough parameters were measured: Hm (maximum dough height (mm); Hm′ (maximum height of the gas release curve (mm); Tx (time of porosity (hours); total volume (total volume of gas produced in mL CO_2_), volume of CO_2_ lost (carbon dioxide volume in mL that the dough has lost during proofing), and retention volume (carbon dioxide volume in mL still retained in the dough at the end of the test). The method conforms to the AACC 89-01 [33] standard for the measurement of yeast activity and gas production.

### 2.5. Bread-Making Procedure

Wheat, rye and legume flour blends as shown in Table 1 were used for baking tests mixed with water; the water addition to individual dough mixes ranged from 57.9 to 66.2% (determined by the Mixolab), 2.0% NaCl, and 1.4% dry yeast. The percentages are based on 100% of the flour mixture. All components were kneaded for 3 min at lower speed and for 4 min at higher speed in a spiral kneader type SP 12 D (Diosna Dierks & Söhne GmbH, Osnabrück, Germany). After 40 min of fermentation (35 °C) the samples were baked for 40 min as follows: at 180 °C for 7 min, at 200 °C for 20 min, and at 160 °C for 13 min, with steam (250 mL) (MIWE Condo).

### 2.6. Physical Characteristics of Bread

The bread loaves were cooled at room temperature and analyzed by a Volscan Profiler volume analyzer (Stable Mycrosystems, Surrey, UK) two hours after baking. The following parameters were evaluated: weight of the bread (g), bread volume (mL), specific volume (mL/g), volume yield (mL/100g flour), aspect ratio of a middle slice. The laser step was set to 1 mm and the rotation speed was set to 1 rps. The specific volume was calculated on the bases of loaf volume and mass.

### 2.7. Sensory Evaluation

The organoleptic characteristics of the breads [34] were evaluated using two descriptors: (i) a point system in which the panelists assessed the intensity of the crust color (max. 5 points), the intensity of the crumb color (max. 5 points) and the overall acceptability using a 9-point hedonic scale; (ii) a 100-point system in which the following were evaluated: Overall appearance and shape (1×); Surface and properties of the crust (2×); Sourness and appearance of the crumb (4×); Structure and elasticity of the crumb (4×); Smell and taste (9×). Recalculations were made for the individual properties according to their meaning (indicated in parentheses). A total of 8–13 panelists trained in sensory analysis and with former experience in evaluating bread samples assessed the sensory attributes of the bread samples.

### 2.8. Color of the Breads

Bread products were analyzed by an E-eye analysis using an IRIS VA400 visual analyzer (Alpha M.O.S.) with a charge-coupled device camera. The bread samples were placed in the measurement chamber. The collected color data were represented by IRIS color codes, which encompass 4096 colors. The analyzed data (color spectrum) was obtained as numerical values of signal intensities.

### 2.9. Statistical Analysis

An analysis of variance (ANOVA) and a Fisher’s multiple range test were applied at a significance level of 5% to describe the significance of the differences between the reference and the samples with different levels of legume flours incorporated. All analyses were performed in triplicate and average values were calculated. XLSTAT 2020.5.1 by Addinsoft was used as the statistical analysis software. Linear regression (Microsoft Excel 2010, Microsoft Corporation, Redmond, WA, USA) was used to display the trend line and the calculated correlation coefficient was evaluated at the significance level α = 0.05 (Microsoft Excel 2010).

## 3. Results and Discussion

### 3.1. Dough Properties

#### 3.1.1. Dough Mixing and Pasting Properties Measured by a Mixolab

Bread dough is a viscoelastic material that exhibits an intermediate rheological behavior between that of a viscous liquid and an elastic solid. The viscoelastic network plays a predominant role in both dough machinability and the textural characteristics of the finished bread.

Water absorption (WA) is an important parameter showing the ability of flour compounds to absorb water and form dough of optimal consistency [35,36]. When using a Mixolab the consistency is expressed as torque in Nm (Newton meter). Water absorption, as well as the mechanical properties of dough with added legume flour, are affected by different factors such as dilution of gluten or the characteristics of native starch. In general, substituting wheat flour with legume flour decreases the total amount of gluten, resulting in the formation of a weaker protein network [37].

Blending the wheat flour with legume flour affects the viscoelastic and mixing properties of the dough. Turfani et al. [14] reported an increased WA in blends containing carob flours, whereas it remained similar to that of the wheat control in blends with lentil flour, which is rich in proteins, but only contains a small amount of soluble fiber (2.9%, slightly more than wheat flour) and about 40% starch. The WA increased, however, when soya flour was added which prolonged the dough stability [4]. Dapcevic et al. [38] confirmed that the substituted flours significantly increased the water absorption measured by a Mixolab. The authors [38] found that starch and its state (emulsifying starch, pre-gelatinized starch, hydrolyzed and spray-dried starch) influenced the dough parameters evaluated by a Mixolab. Bourkit et al. [19] report an increase in water absorption with the addition of chickpea, lentil and bean flours. These additions increase dough development time and decreased dough stability. The increased water absorption was attributed to the higher water holding capacity of legume flour, which is associated with the increased total protein and pentosan content. This might result in modifying water distribution and dynamics in the dough [39].

Compared to the control flour WF(50) + RF(50), the water absorption in our samples significantly (*p* < 0.05) increased with the addition of common bean flour (CBF), directly depending on the amount added, so the ability of the beans to increase the absorption capacity of composite flours was confirmed. The significant increase of the WA was also recorded in composite flours with added broad bean flour (BBF), but in this case the amount of the addition was not important. Composite flours with an addition of CBF and BBF therefore needed the highest amount of water to reach the optimal dough consistency. An addition of RLF and ChF slightly reduced the binding of the composite flour, significantly in the case of WF(50) + RF(35) + RLF(15) (Table 4). Experiments by Du et al. [40] confirmed that variation in functional properties among the legume flours can be associated with the varying ratios of protein to starch and other constituents in the flours. The flour from *Phaseolus* legumes exhibited a high water absorption capacity, a high oil absorption capacity, emulsion activities, and emulsion stabilities. Hydro-koloids incorporation also induced benefits in wheat dough behavior during mechanical shearing and thermal treatment resulting in a significantly increased water absorption, dough development time and stability during mixing and decreased dough weakening with heating [31,37].

Regarding the dough development time (DDT) and dough stability, the additions of legume flour did not considerably affect them. DDT ranged in very close values from 1.5 min in flour WF(50) + RF(35) + RLF(15) to 3.5 min in WF(50) + RF(50). Compared to WF(50) + RF(50), the time required to form dough with the needed consistency was shorter in composite flours with ChF and RLF. In other flours with a proportion of legume flour, DDT remained unchanged. Turfani et al. [14], however, observed that flours from legumes increased DDT (carob flours more than lentil flour) and lentil flour strongly decreased stability due to weakening of the gluten network.

The stability of our doughs was relatively short (1.5 to 3.5 min), including the control flour, because we worked with wheat-rye flour, which has a lower stability due to the content of RF than it would have with pure WF. During dough development, pentosans disturb the protein network formation and influence the dough properties. In wheat flours, dough formation is mainly achieved by hydration of glutenin and gliadin proteins and the disulfide bond formation between them. In case of rye types, the lower protein content requires less time for hydration in spite of the presence of dietary fiber. The rye flours are unable to form a continuous gluten network, therefore rye flour and whole grain rye flour have low DDT and stability values [41]. Voicu et al. [6] noticed a downward trend in the softening degree of the dough with a higher content of rye flour, and in its elasticity.

A positive and expected benefit of replacing a certain proportion of rye flour with legume flour would be to improve DDT and the dough stability. The results, however, showed that none of the additives (legume flours), replacing the proportion of RF in composite flours, had a potentiating effect on the stability of the dough, nor did they improve the pasting properties of the dough by the input of protein structures.

By means of the Mixolab “Chopin+” protocol it is possible to record the mechanical changes due to mixing and heating, simulating the mechanical work, as well as the heat conditions that might be expected during the baking process. The typical Mixolab curve can distinguish different pertaining stages of the dough changes due to both the mixing force and the temperature. During initial mixing, the distribution of the material, the disruption of the initially spherical protein particles and the hydration of the flour compounds occur together with the stretching and alignment of the proteins, leading to the formation of a three-dimensional viscoelastic structure with gas-retaining properties, where the polymeric proteins play the main role [31]. During heating, the native protein structure is destabilized and, as the temperature increases, the role of the proteins becomes secondary, the starch gelatinization being mainly responsible for further torque variations. A decrease in C2 can be the consequence of impediments in the protein unfolding. Conversely, higher C2 values will be reached when the protein network holds the dough structure. When hydrated doughs are heated above a characteristic temperature, the temperature-induced swelling and amylose leaching lead to the formation of viscous pastes [42].

The torque of all evaluated samples started at the optimal value of the dough consistency (1.100 ± 0.095 Nm) and in individual indicative parameters (C1, C2, C3, C4 and C5) it changed depending on the composition of the composite flour. At the point C1, the dough is able to resist the deformation for a certain time, which determines the dough stability [42]. The parameters’ alpha slope, C2, and (C1.2–C2) were recorded when heating reached 52–57 °C, the starch started to gelatinize, and the proteins underwent changes in their quaternary, tertiary, and secondary structures due to protein denaturation [28].

**Figure 1:**
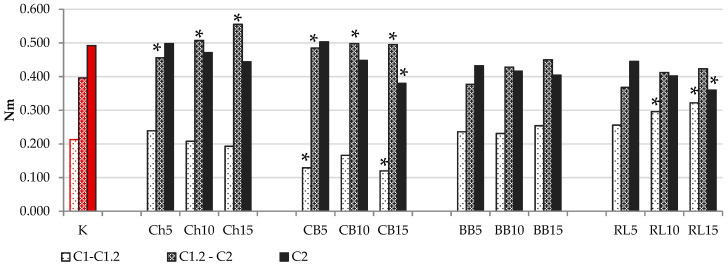
C2 Minimum consistency (Nm). [C1–C1.2] Weakening based on mechanical work at 30 °C. [C1.2–C2] Protein weakening based on temperature increase. * values are significantly different (*p* < 0.05) compared to control. K = WF(50) + RF(50); Ch5 = WF(50) + RF(45) + ChF(5); Ch10 = WF(50) + RF(40) + ChF(10); Ch15 = WF(50) + RF(35) + ChF(15); CB5 = WF(50) + RF(45) + CBF(5); CB10 = WF(50) + RF(40) + CBF(10); CB15 = WF(50) + RF(35) + CBF(15); BB5 = WF(50) + RF(45) + BBF(5); BB10 = WF(50) + RF(40) + BBF(10); BB15 = WF(50) + RF(35) + BBF(15); RL5 = WF(50) + RF(45) + RLF(5); RL10 = WF(50) + RF(40) + RLF(10); RL15 = WF(50) + RF(35) + RLF(15).

In composite flours with an addition of legume flour, except for the lowest addition of ChF and CBF, the C2 value was lower than in the control sample, meaning that the legume flour weakened the protein network in the doughs. The level of protein weakening depended on the amount of legume flour added and on the specific legume flour. The most stable protein network was found with lower additions (5%) of ChF and CBF. In the samples WF(50) + RF(35) + RLF(15) and WF(50) + RF(35) + CBF(15), it was reduced by up to 31.4%, and 27.6% respectively, in comparison to the control sample, which is a considerable reduction. However, if we take into account the calculated value (C1.2 (torque at the end of the holding time at 30 °C)—C2), the effect of legumes on the thermal stability of the protein would be shown more precisely, as the weakening based on mechanical processing at 30 °C would be considered. The higher the value of C1.2–C2, the lower the thermal stability, and in this respect it would be possible to evaluate the additions of BBF and RLF as the most suitable in terms of thermal stability of the protein network in composite flour with an addition of legumes. Statistical differences were confirmed in these samples compared to the control (*p* < 0.05), and the additions of ChF and CBF were significantly less stable. Weakening based on mechanical processing at 30 °C was the lowest in flours with the addition of CBF and the highest in flours with RLF, especially at higher additions (Figure 1).

In flours with the addition of legume flour, especially in the case of higher additions, it was the higher proportion of fiber that could have been responsible for the weaker doughs. According to Rosell et al. [42], the incorporation of fiber into the bread dough system significantly obstructs protein association, and effects bread behavior during the cooling and heating processes. For example, the incorporation of sugar beet fiber in the dough matrix stimulates a disruption of the viscoelastic system, resulting in the production of a weaker dough. Furthermore, it competes for water with the available starch, hence influencing gelling and pasting.

**Figure 2:**
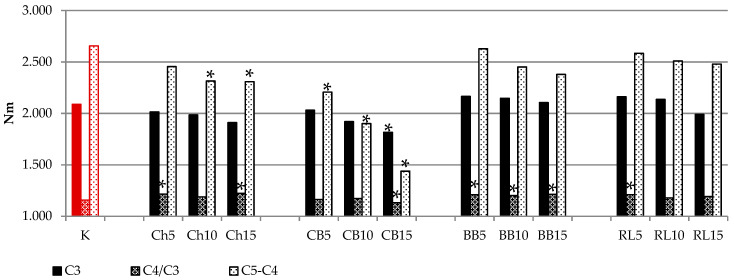
Influence of legume flour addition on torque for starch gelatinization (C3), cooking stability (C4/C3 value) and resistance to retrogradation (C5-C4 value). * values are significantly different (*p* < 0.05) compared to control. K = WF(50) + RF(50); Ch5 = WF(50) + RF(45) + ChF(5); Ch10 = WF(50) + RF(40) + ChF(10); Ch15 = WF(50) + RF(35) + ChF(15); CB5 = WF(50) + RF(45) + CBF(5); CB10 = WF(50) + RF(40) + CBF(10); CB15 = WF(50) + RF(35) + CBF(15); BB5 = WF(50) + RF(45) + BBF(5); BB10 = WF(50) + RF(40) + BBF(10); BB15 = WF(50) + RF(35) + BBF(15); RL5 = WF(50) + RF(45) + RLF(5); RL10 = WF(50) + RF(40) + RLF(10); RL15 = WF(50) + RF(35) + RLF(15).

During the heating stage, doughs containing legume flours showed different pasting properties (C3 value) in comparison to the wheat-rye dough, but we cannot generalize their influence (Figure 2). During this stage, the starch granules absorb the water available in the medium and swell, and the amylose chains leach out into the aqueous intergranular phase promoting an increase of the viscosity and thus a higher torque. These processes continue until mechanical shear stress and temperature limitation lead to the physical disintegration of the granules, which is associated with a decrease in viscosity [31]. In our samples, the starch gelatinization was affected by the legume flour addition; in case of BBF the gelatinization was higher or equal to control in all additions, and in case of RLF only with the additions of 5% and 10%. The addition of CBF and ChF decreased the torque in point C3, with a significant (*p* < 0.05) decrease in case of the addition of 15% of CBF.

A decrease in the temperature resulted in an increase of the torque due to the augmentation of the dough resistance. That increase corresponds to the starch gelation process, in which the amylose chains, which leached outside the starch granules during the heating, are prompted to re-crystalize. The re-association between the starch molecules, especially amylose, leads to the formation of a gel structure. This stage is related to the retrogradation and reordering of the starch molecules. In the cereal slurries, low setback values indicate a low rate of starch retrogradation and low syneresis [43]. The firm gel formed by retrogradation is usually associated with bread staling.

**Figure 3:**
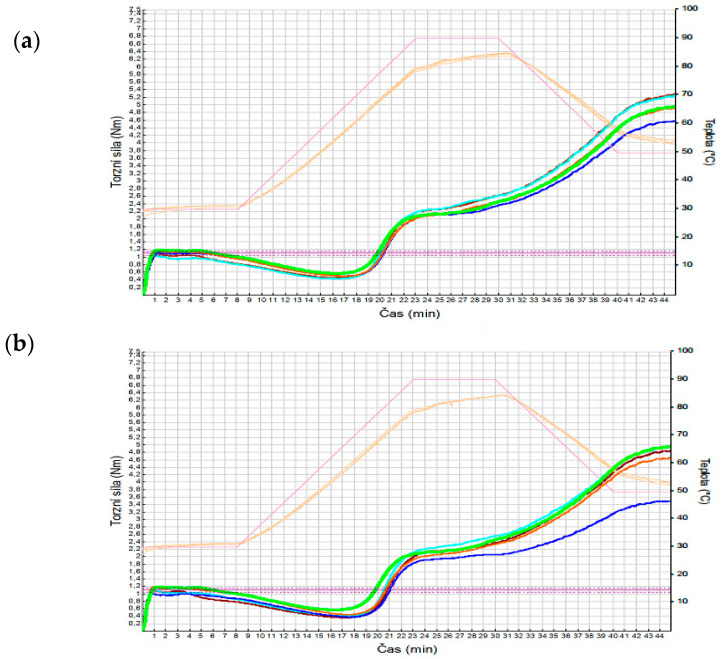
Curves recorded with the Mixolab. (**a**) recorded torque profile in the control sample and samples with an addition of legume flours at an amount of 5%; (**b**) recorded torque profile in the control sample and samples with an addition of legume flours at an amount of 15%; Green—WF(50) + RF(50); Turquoise—addition of BBF; Brown—addition of RLF; Ochre—addition of ChF; Blue—addition of CBF.

Additions of legume flours in composite flours mainly influenced the dough behavior during the cooling phase, as documented in Figure 3a,b. In general, the addition of legume flour did not considerably alter the cooking stability, although some trends were observed for ChF, BBF and RLR, which are related to amylose activity [38]. However, in all samples with CBF there was a significant (*p* < 0.05) increase in resistance to starch retrogradation (Figure 3). The addition of RLF and BBF in composite flours did not have an important effect on retrogradation.

Figure 3a,b show a mixolab curve of the control wheat–rye flour WF(50) + RF(50) (green) and flour with an added proportion of legume flour of 5% (a) and of 15% (b). For the addition of 5% of legume flour, only slight differences were noted compared to the control flour. With a higher addition of 15%, however, the mixolab curve of the composite flour with common bean flour differs considerably, in which different stages can be distinguished with relation to dough changes due to both the mixing force and the temperature. The effect of rye flour is visible on the curves of the composite flours when compared with the mixolab curves of wheat flour [27,35,44], especially the part of the curve between point C3 and C4.

**Figure 4:**
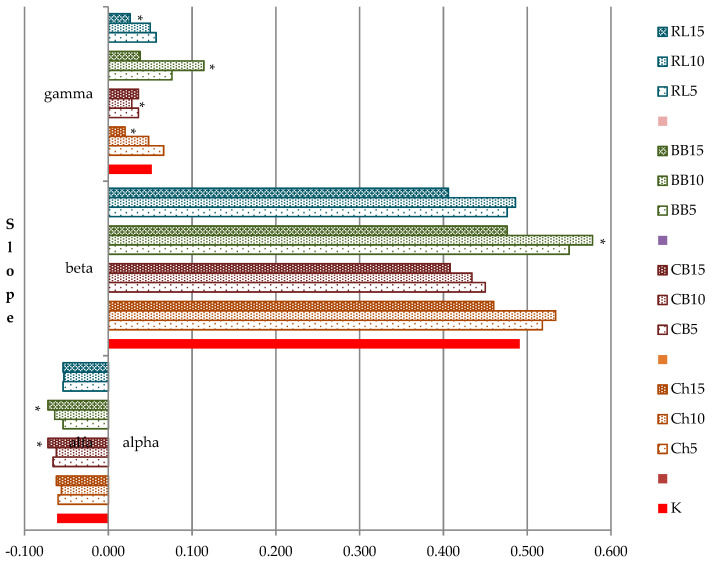
Mixolab calculated parameters alpha, beta and gamma slopes. * values are significantly different (*p* < 0.05) compared to control. K + WF(50) + RF(50); Ch5 + WF(50) + RF(45) + ChF(5); Ch10 + WF(50) + RF(40) + ChF(10); Ch15 + WF(50) + RF(35) + ChF(15); CB5 + WF(50) + RF(45) + CBF(5); CB10 + WF(50) + RF(40) + CBF(10); CB15 + WF(50) + RF(35) + CBF(15); BB5 + WF(50) + RF(45) + BBF(5); BB10 + WF(50) + RF(40) + BBF(10); BB15 + WF(50) + RF(35) + BBF(15); RL5 + WF(50) + RF(45) + RLF(5); RL10 + WF(50) + RF(40) + RLF(10); RL15 + WF(50) + RF(35) + RLF(15).

The presented results are also supported by the data given in Figure 4. The alpha slope, beta slope and gamma slope were calculated of the ascending and descending torques and the angle between ascending and descending curves. In comparison with the control flour, we found significant differences (*p* < 0.05) in the values of alpha slope, beta slope and gamma slope depending on the added legume flour and the amount of its addition. Alpha slope had significantly lower values than the control flour in samples with an addition of 15% of CBF and BBF and higher values with the RLF addition. Samples with added ChF and BBF (5% and 10%) had higher beta slope values than in the control flour (significantly in BBF with an addition of 10%). Compared to the control flour the gamma slope showed significantly lower values with higher additions of ChF, RLF and CBF, and significantly higher values with additions of BBF. It was BBF flour that most significantly affected the observed properties.

The alpha slope shows the protein weakening speed under heating effect, and its values correlate negatively with the rheofermentogram parameters (total volume of gas, retention volume, maximum height of the gas release curve) [27]. Beta slope expresses the speed of starch gelatinization, and the same author [27] found a positive correlation with the bread volume. The level of thermal and enzymatic degradation of starch molecules during the baking process depends on the starch property and the presence of enzymes, i.e., the gelatinization properties of flour. The gamma slope expresses the speed of enzyme degradation. Stanojeska [45] found a positive correlation between maximum viscosity and gamma slope.

**Figure 5:**
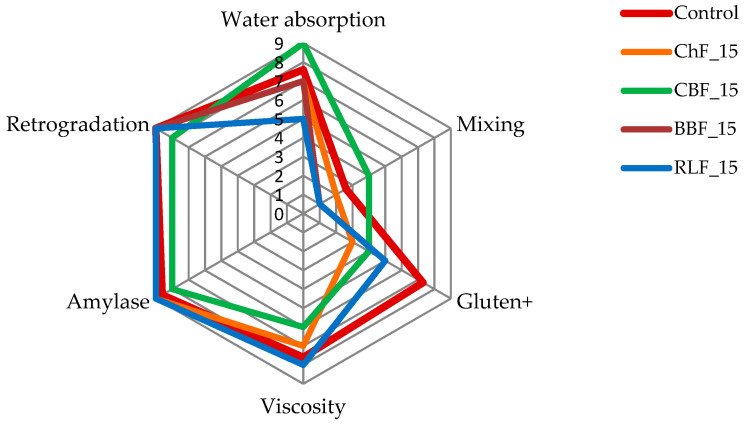
Mixolab Profiler-complete characterization (protein network, starch and enzyme activity) of control flour and flour with legume addition of 15% by fundamental criteria: water absorption index, mixing index, gluten+ index, viscosity index, amylase index and retrogradation index.

Figure 5 (Mixolab Profiler) converts the standard mixolab curve into a set of six scores graduated from 0 to 9 to characterize a flour by fundamental criteria, namely: water absorption index, mixing index, gluten+ index, viscosity index, amylase index and retrogradation index, and serves as a visual aid to indicate the differences between the samples with the highest legume flours and control. In the context of the above, it can be stated that the addition of 15% legume flours caused changes mainly in the WA index, which has already been documented in Table 3, in the mixing index and in the gluten + index, which was documented in Figure 1. The viscosity index, which represents the increase in viscosity during the heating phase, is dependent both on the amylase activity and the starch quality. A lower index, and thus a lower viscosity than for control, is documented in the doughs with ChF and CBF addition.

The dough behavior during mixing, pasting and gelling was influenced by the composition of the composite flour, i.e., the specific legume flour and its amount. The authors also list other possible influences, for example granules of starch having lost their integrity during milling [46,47]. Such damaged starch content influences the water absorption capacity or enzyme accessibility. Miyazaki et al. [48] reported that lower molecular dextrins at 2.5% of substitution retarded the retrogradation of starch in crumbs during storage. In addition, the incorporation of fiber can modify protein–protein interactions as well as both starch gelatinization and gelling processes [42]. The presence of pests in raw materials can also have an uncontrollable effect on the mixolab results. Blandino et al. [49] confirmed a decrease in rheological quality after field damage by cereal bugs, the increasing percentages of damaged kernels leading to a clear decrease in dough stability and protein strength; a significant change in the rheological parameters was noticeable at a 2.5% level of damaged kernels. The particle size of flour can also be a very important factor in determining the properties of the dough. Moreira et al. [50] confirmed the relevant effect of particle size on rheological properties, especially on water absorption and DDT.

According to the results obtained by Mixolab measurements, flours from different raw materials exhibited mixolab profiles which greatly differ from the wheat flour profile [51]. Our flours are not so different from the control wheat–rye flour, and based on the results obtained with the help of a Mixolab, we assume that they can be used to produce bread of good quality thanks to the stated mixing and pasting behavior.

#### 3.1.2. Rheofermentometer Evaluation

Determining the ability to form fermenting gases is crucial in order to produce bread with a good volume. Rheofermentometer analysis of flour and dough enables accurate simulation of processing conditions during production of baked goods containing yeast. The instrument records two curves during the dough fermentation and rising, one describing the development of the dough and another depicting the production and retention of gas.

**Figure 6:**
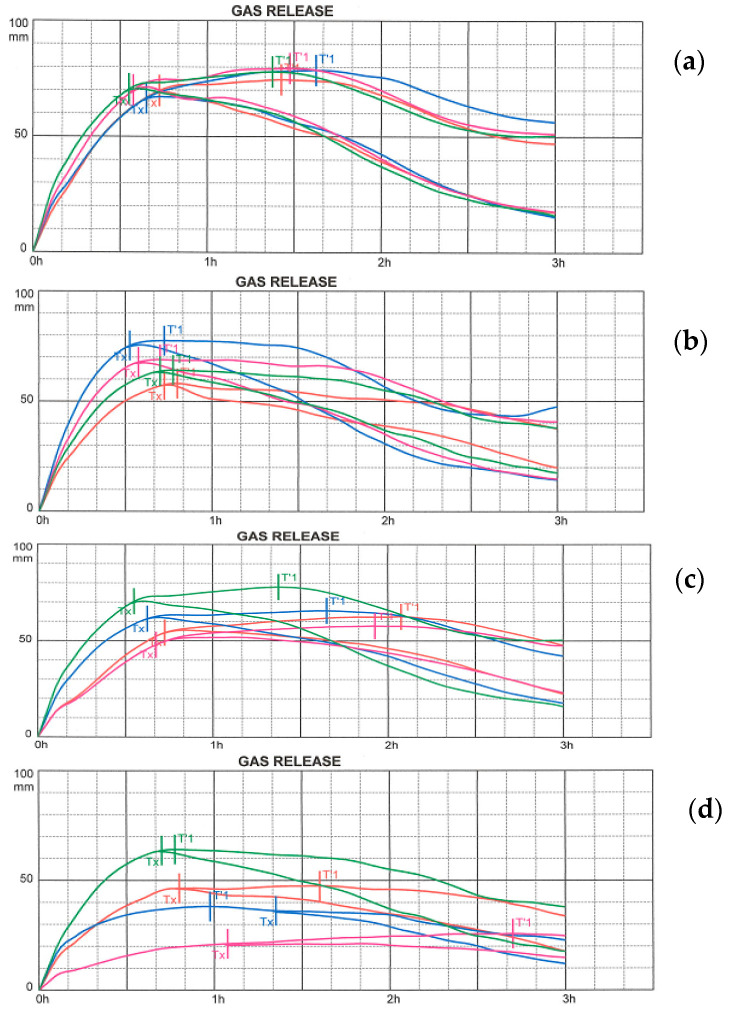
Rheofermentometer curve (**a**) addition of chickpea flour; (**b**) addition of red lentil flour; (**c**) addition of common bean flour; (**d**) addition of broad bean flour. Green—WF(50) + RF(50); Red–addition of 5%; Blue—addition of 10%; Purple—addition of 15%.

Figure 6a–d clearly illustrate the CO_2_ production during the fermentation of doughs with an addition of legume flour. Rheofermentometer curves very similar to those of the control wheat-rye dough were observed with doughs with an addition of chickpea flour and there were no significant differences between these composite flours. The doughs with an addition of broad bean flour had clearly the worst course of the rheofermentometer curves. This was confirmed by a low gas production followed by a low total volume. These flours had a low fermentation capacity, and there were significant differences (*p* < 0.05) between the individual samples caused by the amount of addition. The higher the addition of BBF applied, the lower the total volume, but also the other evaluated parameters.

On the other hand, the addition of 5% and 10% of RLF significantly increased the total CO_2_ volume produced during the analysis, but this gas leaked and the retention volume remained without a significant change in comparison to control.

**Figure 7:**
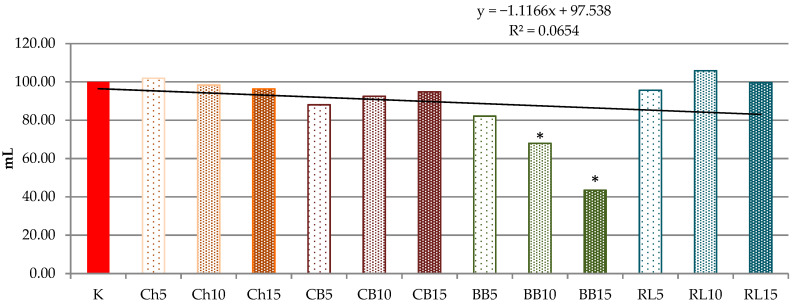
Retention volume—CO_2_ volume in mL still retained in the dough at the end of the test, by Rheofermentometer, calculated in % in comparison to a control sample WF(50)+RF(50). The trend line indicates a trend of decreasing retention volume in doughs with the addition of legume flour. * values are significantly different (*p* < 0.05) compared to control. K = WF(50) + RF(50); Ch5 = WF(50) + RF(45) + ChF(5); Ch10 = WF(50) + RF(40) + ChF(10); Ch15 = WF(50) + RF(35) + ChF(15); CB5 = WF(50) + RF(45) + CBF(5); CB10 = WF(50) + RF(40) + CBF(10); CB15 = WF(50) + RF(35) + CBF(15); BB5 = WF(50) + RF(45) + BBF(5); BB10 = WF(50) + RF(40) + BBF(10); BB15 = WF(50) + RF(35) + BBF(15); RL5 = WF(50) + RF(45) + RLF(5); RL10 = WF(50) + RF(40) + RLF(10); RL15 = WF(50) + RF(35) + RLF(15).

In addition to the amount of CO_2_ produced, the gas retention capacity of dough is also important (Figure 7). In general, the addition of non-wheat raw materials reduces the retention capacity of the dough, but clearly not to the same extent. BBF additions appear to be the least suitable in terms of gas retention capacity of dough, in clear relation to the amount of the addition. For the other legume flours added (with the addition of ChF and RLF), a slight increase in retention was observed compared to the control dough, or a slight decrease (non-significant). Compared to the control, there was a significantly lower (*p* < 0.05) retention volume for doughs with higher additions (10 and 15%) of CBF and BBF. It is evident that three out of the four examined flour mixtures tested by the Rheofermentometer showed similar properties and overall the additives, with the exception of BBF, can be assessed based on the results of this analysis as potentially suitable and applicable in bakery technology.

The bread volume tends to increase when more CO_2_ is produced during fermentation. While a critical CO_2_ volume is noted, the additional gas likely renders the dough matrix unstable due to disproportionation and coalescence. By progressive coalescence and the mechanical destabilization of the gluten network, CO_2_ as well as evaporating water and ethanol escape during baking and result in a reduction of the specific bread volume [52]. These findings have been confirmed and will be evaluated in the following section. Our previous findings regarding the correlations between the production of gas due to the yeast action and the subsequent properties of the experimental bread were confirmed in connection with the application of milk thistle flour, spelt flour, buckwheat flour and others [30,53].

The main problem in applying non-wheat flour to dough is a disturbance of the starch–gluten matrix. This is likely to worsen the gas retention (due to the diluting effect), leading to the observed reduction in the specific bread volume and porosity, and to an increase in the hardness and chewability of the crumbs [54]. Another reason why the rheological behavior of the dough changes is fiber, of which the white bread contains only 2–3% on a dry matter basis [55]. As to the application of legumes, these purposefully increase the fiber proportion, and the effect on the rheological behavior of doughs can be attributed to interactions between the fiber structure and wheat proteins [54].

Babin et al. [56] concluded that the gas retention capacity is less affected by the dough composition than by the present aeration status. In their study, computer-aided simulations of the final loaf volume revealed a linear correlation with the amount of gas in the dough samples. However, when the gas volume fraction exceeded 65%, the simulated volume of baked goods differed from that in the actual products. This result is associated with uncontrolled gas leakage when exceeding the critical CO_2_ volume, which induces disruption of the membranes between individual gas bubbles. Such a mechanism could have been responsible for the results related to the volume of CO_2_ loss. In the case of our samples the amount of losses was positively correlated with gas production (*r* = 0.947*), i.e., at a higher gas production there was also a higher volume of CO_2_ lost. Such a dependence was also found in the case of total volume gas production and retention volume (*r* = 0.972*).

The rheofermentometer curve and measured parameters indicated that, for the doughs with an addition of legume flours, the fermentation completely retained on average 78.1% of the total CO_2_ produced. The impact of various gas production rates on the time of dough porosity Tx and the corresponding CO_2_ volume were studied by Verheyen et al. [52]. They evaluated rheofermentometer data for the prediction of baking performance. In our samples the time of porosity (gas starts to escape the dough matrix) was prolonged by the addition of legume flours, meaning that the gas losses started later, but only in the case of doughs with higher BBT additions (10%, 15%) was this a significant prolongation. With relation to the final retention volume, this did not appear to be essential as evidenced by the retention coefficient.

The main yeast substrates in wheat flour dough are fructose, glucose, sucrose, and maltose. The content of these fermentable sugars is continuously modified throughout the technological process due to both their amylolytic production and yeast consumption during the fermentation process. Codina et al. [57] assessed the correlation between the fermentable sugar content of dough and its behavior during fermentation, and found out that the gas production showed a positive correlation with the glucose content after 60 min of fermentation (*r* = 0.846) and a negative correlation with the fructose content after 120 min of fermentation (*r* = −0.993). Dough density after kneading depends on the type of yeast and the number of colony forming units were studied by Verheyen et al. [52], who found that the fermentation behavior was similar, and detected only small variations in the final bread volume irrespective of yeast concentration or duration of fermentation.

The results of the evaluation using a Rheofermentometer indicated that the weakest flours in terms of the ability to create and maintain the largest possible volume of gases in the dough were samples with the addition of BBF, depending on the amount of the addition. This was also confirmed by a bakery experiment in which it was mainly these flours which produced low-volume bread.

### 3.2. Bread Evaluation

Basically, the process involved in the production of bread is a highly complex one that entails a range of parameters which have to be regulated [21]. The role of gluten and starch in bread making has already been well investigated, and it is precisely gluten, in reality, that is the skeleton of wheat flour dough, and plays an important role in gas retention for making light, leavened products [29,35,37,38]. Additions of non-wheat flour cause dilution and subsequent weakening of the wheat dough.

**Figure 8:**
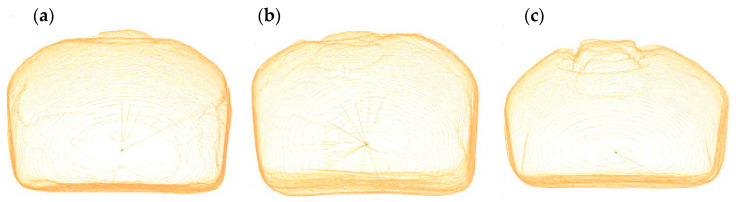
Bread volume and bread aspect ratio by Volscan profiler. (**a**) control wheat-rye bread; (**b**) bread with RLF 5% (bread with the addition of legume flour with the highest volume); (**c**) bread with BBF 15% (bread with the addition of legume flour with the lowest volume).

Our results of the evaluation of dough prepared from wheat–rye flour and from composite flours with an addition of legume flour, which to some extent replace rye flour in the recipe, give indications that these composite flours are suitable for bread production. It is clear from Figure 8 that the differences between the control bread and the bread with the highest measured volume and the lowest measured volume are not large, and all breads are technologically acceptable.

Turfani et al. [14] confirmed that the blending of wheat flour with 5–6% of legume flour generally did not alter the loaf volume. However, increasing the amount of legume flour to 10–12% or 24% reduced the loaf volume. The final bread volume depends on the dough expansion during fermentation and baking, and the ability of the matrix to stabilize the retained gas. In all our samples with additions of legume flour, the bread volume was significantly lower compared to wheat–rye breads (*p* < 0.05). The smallest reduction was in breads with the addition of RLF and ChF. When compared with the prediction of bread volume based on data from a Rheofermentometer, we can conclude that the bread volume correlated (α = 0.05) with the retention volume measured by a Rheofermentometer (*r* = 0.643*). This correlation was higher than the correlation with the total volume measured by a Rheofermentometer and volume CO_2_ lost (*r* = 0.442; *r* = 0.549* respectively).

**Figure 9:**
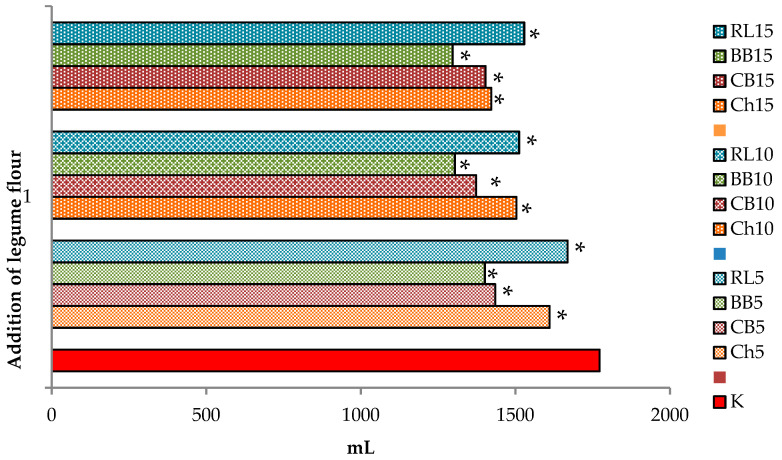
Bread volume of trial breads. Comparing breads with the addition of 5%, 10% and 15% of legume flour. * values are significantly different (*p* < 0.05) compared to control. K = WF(50) + RF(50); Ch5 = WF(50) + RF(45) + ChF(5); Ch10 = WF(50) + RF(40) + ChF(10); Ch15 = WF(50) + RF(35) + ChF(15); CB5 = WF(50) + RF(45) + CBF(5); CB10 = WF(50) + RF(40) + CBF(10); CB15 = WF(50) + RF(35) + CBF(15); BB5 = WF(50) + RF(45) + BBF(5); BB10 = WF(50) + RF(40) + BBF(10); BB15 = WF(50) + RF(35) + BBF(15); RL5 = WF(50) + RF(45) + RLF(5); RL10 = WF(50) + RF(40) + RLF(10); RL15 = WF(50) + RF(35) + RLF(15).

Figure 9 shows that the addition of legume flour led to a decrease in bread volume. With the addition of 5% of legume flour, the best bread volume result was obtained by applying RLF and then ChF, and the smallest volumes were observed with the addition of CBF and BBF. With the addition of 10% of legume flour the best bread volume results were obtained by applying RLF followed by ChF, and the smallest volumes were observed with the addition of CBF and BBF. With the addition of 15% of legume flour, the best bread volume result was obtained by applying RLF, followed by additions of ChF and CBF, and the smallest volume was observed with the addition of BBF. These results are consistent with other previously found data from a Mixolab and Rheofermentometer. RLF proved to be the most suitable legume flour in terms of bread volume, even with the highest selected addition of 15%. The least suitable legume flour was BBF, in all additions, despite the fact that the Mixolab showed good thermal stability and good porosity properties in doughs with the addition of BBF, but there were already indications of certain shortcomings in the evaluation of the gamma slope and beta slope.

Other bread parameters were also affected by the addition of legume flour, e.g., the specific bread volume and the aspect ratio of a middle slice, which expresses cambering, and for which the same trend was found as for bread volume.

Based on a comprehensive evaluation of the physical properties of the trial breads, we can conclude that, although differences between the samples were found, all breads have properties that are acceptable.

In addition to technological properties, sensory properties are also important, which ultimately determine the final quality and acceptability of the product. The trial breads from wheat–rye flour, or with legume flour added in a proportion of 5%, 10% and 15% instead of rye flour, were subjected to a sensory evaluation. Only based on this parameter it is possible to present the final conclusions.

**Figure 10:**
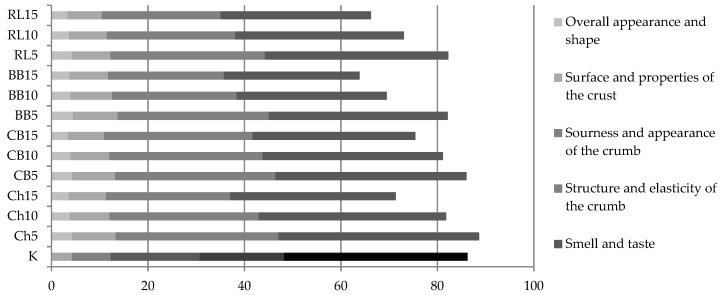
Sensory evaluation of the trial breads by means of a 100-point system. K = WF(50) + RF(50); Ch5 = WF(50) + RF(45) + ChF(5); Ch10 = WF(50) + RF(40) + ChF(10); Ch15 = WF(50) + RF(35) + ChF(15); CB5 = WF(50) + RF(45) + CBF(5); CB10 = WF(50) + RF(40) + CBF(10); CB15 = WF(50) + RF(35) + CBF(15); BB5 = WF(50) + RF(45) + BBF(5); BB10 = WF(50) + RF(40) + BBF(10); BB15 = WF(50) + RF(35) + BBF(15); RL5 = WF(50) + RF(45) + RLF(5); RL10 = WF(50) + RF(40) + RLF(10); RL15 = WF(50) + RF(35) + RLF(15).

The sensory evaluation showed that the breads were characterized by a regular shape, a slight surface roughness and a crust color ranging from light yellow to light brown. The results of the evaluation by means of a 100–point system are shown in Figure 10. From a sensory point of view compared to wheat-rye bread, bread with 5% of CBF was at the same level. The bread with the addition of 5% of ChF was evaluated as even better. Seven variants of the additions out of a total of 16 scored more than 80 points (out of 100). Only three variants scored below 70 points, namely bread with the addition of 10% and 15 % of BBF, and bread with the addition of 15% of RLF. None of the experimental variants scored below 60 points, which we would rate as a poor sensory quality.

The extent to which bread will receive a positive sensory evaluation is largely determined by its smell and taste, and it is for these criteria that the ratings of breads with legume flour were the best. The aromas and flavors of the added non-cereal ingredients have improved the sensory profile of wheat-rye bread, which we clearly consider a benefit. In the case of ChF and CBF this was up to an addition of 10%, and in the case of RLF and BBF up to an addition of 5%. The other variants had an aroma and taste that was too pronounced or foreign, and were rated as worse compared to the control bread. The worst rated bread was with the addition of 15% of BBF (28.1 points in comparison to 38.5 points obtained by wheat–rye bread, or to 41.6 points obtained by the bread with the addition of 5% of ChF, which was the best rated aroma and taste).

These findings were not surprising, because the addition of non-cereal raw materials, apart from nutritional benefits, also brings interesting sensory properties. Lower additions in general keep the quality attributes of conventional bread. Sabanis and Tzia [4] indicate that the incorporation of non-bakery raw materials (rice, corn, soy) in bread wheat flour up to a level of 10% results in bread without any negative effect on quality attributes such as color, hardness, and flavor and their reasonable acceptance offers a promising nutritious and healthy alternative to consumers. As the level of the addition of increases above 20%, bread quality characteristics deteriorate proportionally (low loaf volume, lack of flavor, black specks, coarse crumb, and a hard texture) as a result of the replacement of gluten by the added protein. Notwithstanding the different machinability and handling, the results of the experimental mixtures can result in bread with an acceptable taste and texture, without an excessive legume flavor [14]. Bread made with blends containing legume (10–15%) has an improved nutritional composition as well as appreciated technological and sensorial traits. Beyond 15% supplementation, additives incorporation might be a good alternative to compensate for the dilution of gluten, thereby maximizing nutritional advantages and overcoming technological flaws [19].

**Figure 11:**
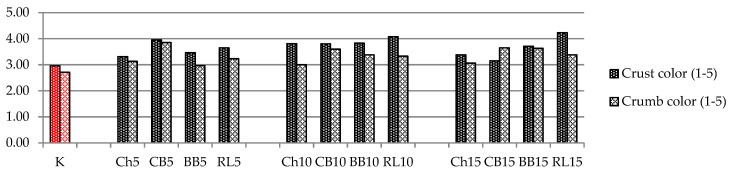
Intensity of the crust and crumb color rated by a 5-point system, 1 = least intensive color, 5 = most intensive color. K = WF(50) + RF(50); Ch5 = WF(50) + RF(45) + ChF(5); Ch10 = WF(50) + RF(40) + ChF(10); Ch15 = WF(50) + RF(35) + ChF(15); CB5 = WF(50) + RF(45) + CBF(5); CB10 = WF(50) + RF(40) + CBF(10); CB15 = WF(50) + RF(35) + CBF(15); BB5 = WF(50) + RF(45) + BBF(5); BB10 = WF(50) + RF(40) + BBF(10); BB15 = WF(50) + RF(35) + BBF(15); RL5 = WF(50) + RF(45) + RLF(5); RL10 = WF(50) + RF(40) + RLF(10); RL15 = WF(50) + RF(35) + RLF(15).

In terms of the intensity of the crust and crumb color and compared to the control bread (Figure 11), the additions of legume flour caused darker colors, which might be positive from a marketing point of view, as there is interest in darker breads among consumers.

**Figure 12:**
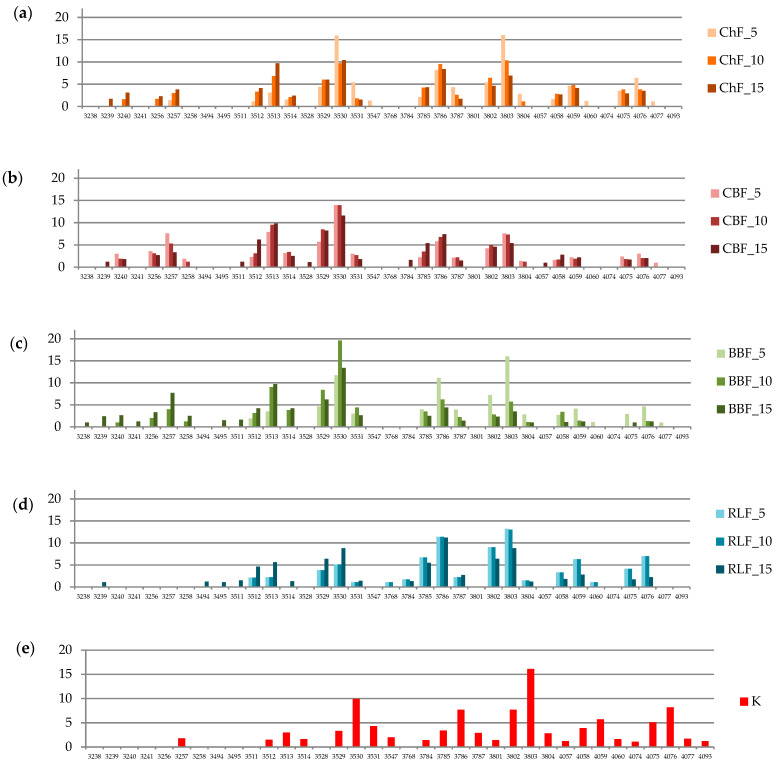
Color spectra by E-eye. (**a**) bread with addition of chickpea flour; (**b**) bread with addition of common bean flour; (**c**) bread with addition of broad bean flour; (**d**) bread with addition of red lentil flour; (**e**) control bread. K = WF(50) + RF(50); ChF_5 = WF(50) + RF(45) + ChF(5); ChF_10 = WF(50) + RF(40) + ChF(10); ChF_15 = WF(50) + RF(35) + ChF(15); CBF_5 = WF(50) + RF(45) + CBF(5); CBF_10 = WF(50) + RF(40) + CBF(10); CBF_15 = WF(50) + RF(35) + CBF(15); BBF_5 = WF(50) + RF(45) + BBF(5); BBF_10 = WF(50) + RF(40) + BBF(10); BBF_15 = WF(50) + RF(35) + BBF(15); RLF_5 = WF(50) + RF(45) + RLF(5); RLF_10 = WF(50) + RF(40) + RLF(10); RLF_15 = WF(50) + RF(35) + RLF(15).

The color spectrum by E-eye was also measured and converted to numerical values of signal intensities. The spectra of breads with the addition of individual legumes and control bread are shown in Figure 12a–e. Darker colors were found in breads with the addition of BBF, CBF and ChF. The RLF addition had the least colors in the dark spectra, and did not differ from the control sample for other colors. No such finding was found in the sensory evaluation of the color intensity. Statistically significant differences (*p* < 0.05) in colors were found, which occurred in all samples between their signal intensity in the control bread and bread with the addition of legume flours. Of the darker bread colors, colors with codes 3529 and 3530 were present in the control bread, but in breads with BBF and CBF, the intensity of these colors was significantly higher. Another group of lighter colors (3082–3804) was already significantly lower in breads with the addition of BBF and CBF compared to the control bread, and such a trend in the spectrum of lighter colors typical of ordinary bread was also found in other cases. In particular, bread with CBF had a considerably lower proportion of lighter bread colors. In the case of bread with RLF, the light bread color 3785 was significantly more important compared to the control bread, but for most colors there were no considerable differences between the control bread and bread with RLF.

Although it is a very challenging task to mimic the unique bread making properties of wheat flour, it is possible to create products having a similar behavior to wheat flour dough and bread, but with improved functional properties [35]. The nutritional benefits of applying legumes to bread as a product of massive consumption are known, but the problem is the presence of antinutrients, such as trypsin inhibitor, lectin, a-amylase inhibiting factor, goitrin, and others [28]. Therefore, the question is whether the baking time of bakery products is sufficient for an effective inactivation of these compounds. A solution to reduce the risk could be a legume flour pre-treatment (e.g., germination and fermentation) [19]. Despite the risk, the nutritional value and the technological properties of legumes are useful for increasing the nutritional and functional value of wheat or wheat–rye bread.

## 4. Conclusions

The use of non-bakery raw materials as an addition to wheat flour is becoming increasingly popular, mainly due to the nutritional benefits of different plant resources. On the other hand, these non-bakery raw materials change the technological parameters of the flours, doughs, and final products, mostly in a negative way. The results of the present study suggest that the incorporation of legume flours influences considerably the evaluated properties, and those flours with the addition of legumes behaved differently than wheat–rye flour. The properties of the final products (breads) also changed, both objectively and from a sensory point of view. It could be concluded that:

- water absorption significantly increased with the addition of common bean flour (CBF) and broad bean flour (BBF), and an addition of red lentil flour (RLF) and chickpea flour (ChF) slightly reduced the binding of the composite flour; none of the additives (legume flours), replacing the proportion of rye flour (RF) in composite flours, had a potentiating effect on the stability of the dough;
- the mixolab curve can distinguish different stages pertaining to the dough changes due to both the mixing force and the temperature, and the results showed a weakening of the protein network depending on the amount of legume flour added and on the specific legume flour. In terms of the interaction of mechanical processing and thermal procedure, the most stable (at the level of the control wheat–rye flour) was flour with the addition of CBF, in all applied proportions of this addition;
- the addition of legume flours mainly influenced the dough behavior during the cooling phase, and in all samples with a higher proportion of legume flour there was an increase in resistance to starch retrogradation. From the evaluated samples, the additions of CBF had the biggest impact on increasing the resistance to retrogradation;
- in comparison with the control flour, significant differences were found in the values of the slopes alpha, beta and gamma depending on the added legume flour and the amount of its addition. BBF addition most significantly affected the observed properties;
- determining the ability to form fermenting gases is crucial in order to produce bread with a good volume, and the doughs with an addition of BBF clearly had the worst course of the rheofermentometer curves. The other three examined flour mixtures showed similar properties and overall the additives, with the exception of BBF, can be assessed as potentially suitable and applicable in bakery technology on the basis of the results of this analysis;
- the bread volume depends on the dough expansion during fermentation and baking, and the ability of the matrix to stabilize the retained gas. In all our samples with additions of legume flour, the bread volume was significantly lower compared to wheat–rye breads. RLF proved to be the most suitable legume flour in terms of bread volume, even with the highest selected addition of 15%. The least suitable legume flour was BBF, in all additions;
- the sensory evaluation confirmed that the addition of legume flour of 5% gives breads rated approximately the same or even better than the control bread; higher additions (15%) add extra typical legume flavors and aromas to breads, which have been assessed less positively, especially in the case of BBF;
- the effect of individual additions on crust and crumb color was significant, which was also confirmed by the E-eye color spectrum; breads with the addition of ChF, CBF and BBF had a considerable proportion of darker colors compared to the control bread and bread with RLF.

In terms of dough formation and its mechanical properties, based on a comprehensive evaluation, it is possible to consider as the best flours those with the addition of CBF. Regarding the formation and retention of fermentation gases, flour with the addition of RLF was determined to be the most suitable, which was also confirmed by a baking experiment and the volume of bread. From the point of view of sensory evaluation, even with higher additions, breads with the addition of CBF were evaluated positively, but the additions of other legume flours at lower proportions were also approximately at the level of a control. In general, the least suitable addition was BBF.

## Figures and Tables

**Table 1:** Quality parameters of commercial flours.

	WF	RF	ChF	BBF	CBF	RLF
Energy value (kJ)	1464	1396	1322	1300	1354	1263
Carbohydrate content (g/100 g)	71	68	59	49.7	49	54
of which sugars	1.59	3	-	3.3	3.2	2
Fiber content (g/100 g)	3.3	11	-	-	-	-
Protein content (g/100 g)	11	7.5	16.7	28	25	17
Fat content (g/100 g)	1.3	1.1	4.8	2.5	1.2	1.5
of which saturated	0.3	0.2	0.5	0.4	0.5	0.5
Salt (g/100 g)	0.01	0.01	0.07	0.01	0.01	0.01

WF (wheat flour), RF (rye flour), ChF (chickpea flour), BBF (broad bean flour), CBF (common bean flour), RLF (red lentil flour).

**Table 2:** Combinations of evaluated composite flours (experimental design).

Chickpea Flour	Broad Bean Flour	Common Bean Flour	Red Lentil Flour
WF(50) + RF(50)	WF(50) + RF(50)	WF(50) + RF(50)	WF(50) + RF(50)
WF(50) + RF(45) + ChF(5)	WF(50) + RF(45) + BBF(5)	WF(50) + RF(45) + CBF(5)	WF(50) + RF(45) + RLF(5)
WF(50) + RF(40) + ChF(10)	WF(50) + RF(40) + BBF(10)	WF(50) + RF(40) + CBF(10)	WF(50) + RF(40) + RLF(10)
WF(50) + RF(35) + ChF(15)	WF(50) + RF(35) + BBF(15)	WF(50) + RF(35) + CBF(15)	WF(50) + RF(35) + RLF(15)

**Table 3:** Instrumental settings defined in the Mixolab for running the samples.

Mixing Speed	80 rpm
Target torque	1.10 Nm
Dough weight	75 g
Tank temperature	30 °C
Temperature 1st step	30 °C
Duration 1st step	8 min
1st temperature gradient	4 °C/min
Temperature 2nd step	90 °C
Duration 2nd step	7 min
2st temperature gradient	−4 °C/min
Temperature 3rd step	50 °C
Duration 3rd step	5 min
Total analysis time	45 min

**Table 4:** Effect of legume flours on water absorption.

Legume Flour Level (%)	WA (%)	Legume Flour Level (%)	WA (%)
0	62.4 ± 0.40 b	0	58.9 ± 0.28 b
Chickpea flour		Red lentil flour	
5	61.0 ± 0.39 a	5	58.7 ± 0.28 b
10	61.1 ± 0.48 ab	10	58.8 ± 0.28 b
15	61.6 ± 0.39 ab	15	57.4 ± 0.35 a
Common bean flour		Broad bean flour	
5	63.6 ± 0.42 c	5	60.1 ± 0.28 c
10	64.7 ± 0.35 c	10	60.2 ± 0.35 c
15	66.2 ± 0.39 d	15	60.1 ± 0.39 c

Values followed by the same letter in the column are not significantly different (*p* > 0.05).

## Data Availability

Not applicable.
